# Hemipelvectomy and 3D Custom-Made Prosthesis Implantation: Early Surgical, Radiographic, and Functional Results—A Multicentre Study

**DOI:** 10.3390/medicina62050951

**Published:** 2026-05-13

**Authors:** Grzegorz Guzik, Daniel Pyrka, Paweł Łęgosz, Piotr Szremski, Piotr Biega

**Affiliations:** 1 Department of Orthopaedic Oncology, Subcarpatian Oncology Centre, Dworska 77A, 38-420 Korczyna, Poland; 2 Department of Trauma and Orthopaedic Surgery, Hospital in Krosno, 38-400 Krosno, Poland; 3 Orthopedics and Traumatology Clinic in Warsaw, Medical University of Warsaw, 02-091 Warsaw, Poland; 4 Trauma and Orthopaedic Department, District Hospital in Przemyśl, 37-700 Przemyśl, Poland

**Keywords:** hemipelvectomy, customized implants, pelvic surgery, bone defects, endoprostheses

## Abstract

*Background*: There are still insufficient studies based on large patient cohorts that evaluate both functional and surgical outcomes after internal hemipelvectomy and 3D-printed endoprosthesis implantation. This study aimed to determine how the method of pelvic bone defect reconstruction determines early functional, surgical, and radiographic outcomes. *Methods*: The aims of the study were achieved based on retrospective observations of 49 patients who underwent surgical treatment at several centres in Poland. All patients underwent internal hemipelvectomy and implantation of 3D-printed prostheses. Surgical parameters were assessed, including operative time, blood loss, and surgical complications (infections, implant loosening, dislocations), as well as bone osseointegration. Functional outcomes were assessed using the HHS and MSTS-93 scales, and pain intensity was measured using the VAS. Outcomes were stratified according to implant design and fixation method based on the West China Classification. *Results*: The most commonly performed procedures were internal hemipelvectomies of Enneking Type I + II and II + III, with reconstructions most frequently classified as WChC-Aa (15%) and WChC-Bb (44%). Functional assessments revealed significant improvement across all patients. Both the HHS and MSTS-93 values demonstrated marked progress from preoperative averages of 44 (HHS) and 12 (MSTS-93) to 64, 70.2, and 76 (HHS) and 19, 20, and 20.2 (MSTS-93) after 6 weeks, 3 months, and 6 months, respectively. Correspondingly, pain intensity decreased from a mean preoperative VAS score of 8.5 to 4.4, 3.4, and 3.2 after surgery. Osseointegration occurred in 53%, 75%, and 83% of patients after 6 weeks, 3 months, and 6 months, respectively. Wound-healing complications were observed in 6 patients, while deep infection developed in 4 cases. In 3 patients, the implant was removed. Implant loosening was noted in imaging studies in 7 patients (14%) and 8 patients (16%) at 3 and 6 months postoperatively, respectively. Local tumour recurrence was observed in 6 cases. *Conclusions*: The extent of pelvic tumour resection and the reconstruction method appear to influence surgical parameters, the risk of complications, and operative time. Functional outcomes measured using VAS, HHS, and MSTS-93 scales showed improvement following surgical treatment and tended to improve over time; however, these findings should be interpreted with caution given the relatively short follow-up period and the lack of assessment of minimal clinically important difference (MCID). The use of 3D-printed implants may facilitate precise pelvic reconstruction and enable early mobilization and rehabilitation.

## 1. Introduction

Primary and metastatic bone tumours involving the axial skeleton pose a substantial therapeutic challenge. Both oncological and orthopaedic treatments are complicated and associated with unsatisfactory outcomes. Cartilaginous tumours—both benign and malignant—are particularly common in the pelvis, whereas Ewing sarcomas and osteosarcomas occur less frequently. Metastatic lesions from renal, breast, and thyroid cancers, as well as multiple myeloma, are also frequently observed [1,2].

Surgical treatment of malignant primary bone tumours should be radical. The standard approach involves the removal of the tumour with an adequate margin of healthy tissue (R0 resection), which reduces the risk of local recurrence and metastasis and improves patient survival. In metastatic lesions, radical tumour resection may also improve prognosis by reducing local recurrence and implant loosening rate, as well as the need for adjuvant radiotherapy. Furthermore, it improves patients’ functional status and survival outcomes [1,2].

Pelvic bone tumour resections have been performed for several decades. The Enneking classification of internal hemipelvectomies remains clinically relevant and widely used, although numerous modifications have been proposed [3]. The most frequently performed procedures are Type II internal hemipelvectomies, as well as combined Type II with III or II with I. In these cases, pelvic girdle reconstruction is necessary. Currently, hemipelvectomies involving limb amputation are generally avoided. Pelvic reconstruction is unnecessary in Type I and Type III internal hemipelvectomies [4,5].

Xu et al. proposed an innovative classification system for pelvic bone defect reconstruction following internal hemipelvectomies using 3D-printed pelvic endoprostheses, based on the defect morphology, named the West China Classification. The system categorizes resections as follows: Type A—resection of the acetabulum; Type B—resection of the acetabulum and the sacroiliac joint; and Type C—resection involving the acetabulum, sacroiliac joint, and pubic symphysis. Type A is further subdivided into Aa—sparing the pubic symphysis, and Ab—involving the pubic symphysis. Type B is subdivided into Ba—not involving the obturator foramen, and Bb—involving the obturator foramen [6]. The classification is clinically useful and allows for comparison of treatment outcomes in patients receiving 3D custom-made implants, not only according to the extent of resection but also regarding the structural design of the endoprosthesis.

In the past, various methods were used to reconstruct pelvic bone defects, including allogeneic and autologous bone grafts, bone cement, fixation techniques, and arthrodesis, as well as saddle-type prostheses, ice-cream cone prostheses, and modular implants. However, these approaches were associated with unsatisfactory outcomes and high complication rates [7,8,9,10,11]. Over the past two decades, advances in medical engineering have enabled the increasingly widespread use of patient-specific implants, designed on the basis of preoperative CT imaging and manufactured using 3D-printing technology. Custom-made implants continue to undergo refinement, with successive generations being developed and treatment outcomes showing continuous improvement [12,13,14,15,16].

There are still insufficient studies addressing surgical, radiographic, and functional outcomes after internal hemipelvectomy with 3D custom-made prostheses implantation, and available publications predominantly involve small and heterogeneous patient cohorts. Continued follow-up and evaluation of issues related to the design, implantation, and osseointegration of 3D implants are crucial. Such research may enable further improvements in implant design and contribute to better treatment outcomes [17,18].

The aim of this multicentre study was to evaluate early treatment outcomes in patients who underwent implantation of custom-made prostheses following extensive pelvic bone tumour resection. The analysis included surgical, radiographic, and functional outcomes assessed in relation to the implant design. We hypothesized that the use of 3D-printed custom-made prostheses would lead to favourable functional outcomes and acceptable complication rates, and that outcomes would vary significantly depending on the WChC reconstruction class. Patient data were analysed for up to six months after surgery. This period is considered sufficient to achieve functional osseointegration.

## 2. Material and Methods

Between 2018 and 2024, 66 internal hemipelvectomies were performed across four Polish Orthopaedic Departments specializing in the treatment of primary and metastatic musculoskeletal tumours. The study included 49 patients who, following tumour resection, received custom-made 3D-printed implants. The remaining 17 patients underwent internal hemipelvectomy without reconstruction (8 cases) or with traditional reconstruction methods (9 cases). The study was conducted in accordance with the Strengthening the Reporting of Observational Studies in Epidemiology (STROBE) guidelines. The study protocol was the same in all centres.

This research was performed as a retrospective analysis of anonymized patient data. According to national regulations, it did not require formal ethics committee approval. All the patients provided written consent to participate in this study.

Inclusion criteria were single metastatic lesions with a good life expectancy of more than one year and bone damage not suitable for reconstruction with standard prostheses. Exclusion criteria included patients with a life expectancy of less than one year, Type I and III resections without reconstruction, open hemipelvectomy, paediatric patients, and patients qualified for other surgical treatment methods.

## 3. Patient Qualification and Implant Design

The patients’ surgical treatment qualification was carried out at a multidisciplinary oncological meeting. Oncological and surgical staging and the patients’ treatment options and prognosis were analysed. Based on MRI and CT scans, the extent of tumour resection was established. Medical engineers and orthopaedic surgeons designed the prosthesis with particular attention to the surgical approach and prosthesis stabilisation elements. After two weeks, the prosthesis project was finally accepted by surgeons, and the production process was started. After the next 5–6 weeks, the implant was ready for use.

**Figure 1:**
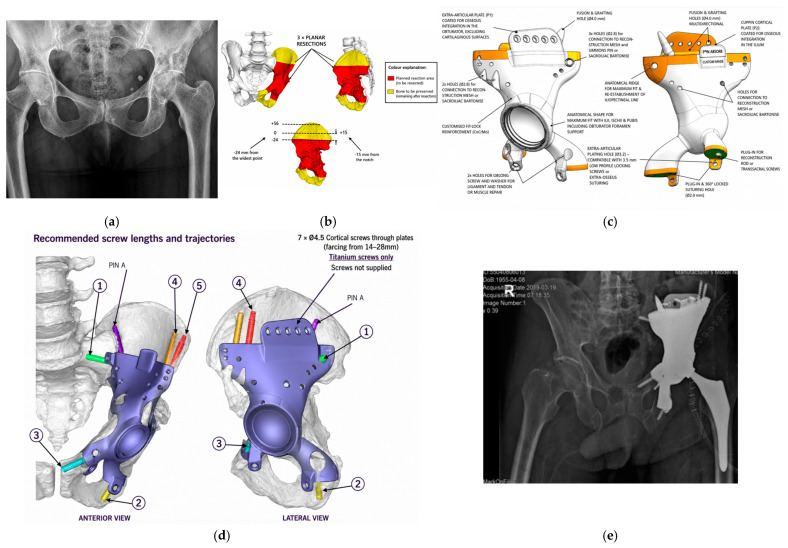
(**a**–**e**) Subsequent steps of implant design and the postoperative radiograph. (**a**) A preoperative radiograph with a visible osteolytic lesion in the acetabular region. (**b**) The pelvic resection extent. (**c**,**d**) A custom Ti-6AI-4V hemipelvic replacement implant. (**e**) A postoperative radiograph of the implanted 3D custom-made prosthesis.

Over the years, the implant projects changed, and morphological reconstruction was abandoned. The focus shifted towards increasing mechanical endurance and osseointegration conditions. To improve the precision of bone cutting and tumour resection, specially designed and manufactured bone-trimming tools were used. Intraoperative navigation systems were not used. The implant development process is presented in Figure 1a–e.

All procedures were performed by experienced surgeons. Most patients were women (31 cases), while men accounted for 18 cases. The mean age was 58 years for women and 62 years for men. The follow-up period ranged from 8 to 64 months, with a mean of 21 months. Patient data were analysed for up to 6 months to achieve the study objectives. Metastatic lesions were caused by the following primary tumours: breast cancer in 19 patients, renal cancer in 5 patients, multiple myeloma in 3 patients, lung cancer in 2 patients, and thyroid cancer in 5 patients. Other causes were identified in 15 patients, including rare cancers and metastases of unknown primary origin (FPI). The patients’ medical records were evaluated with particular emphasis on tumour type, disease duration, and prognosis. Patients’ general condition was assessed, along with pain location and intensity using the Visual Analogue Scale (VAS), functional status according to the Harris Hip Score (HHS), Musculoskeletal Tumour Society (MSTS-93) score, mobility, joint range of motion, and the type of orthopaedic device. No correlation was found between the results and patient comorbidities.

**Figure 2:**
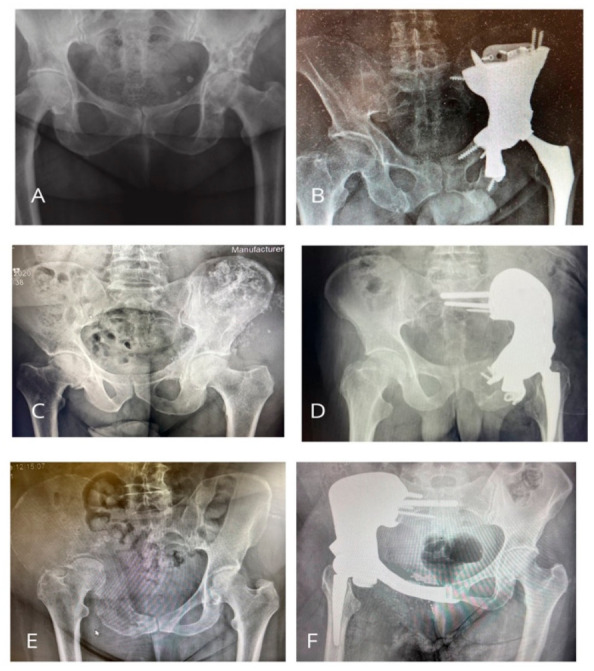
Pelvic bone tumour radiograms and examples of Enneking resection types: (**A**,**B**) breast cancer metastasis and II + III Enneking resections with WChC-Ab reconstruction; (**C**,**D**) thyroid cancer with Type I + II Enneking resections and WChC-Bb reconstruction; (**E**,**F**) breast cancer metastasis with I + II + III Enneking resections and WChC-C reconstruction.

Before the procedure, standard radiographs and a CT scan of the pelvis were performed to accurately assess the size and location of the tumour, the bone tissue condition, and the cortical bone defects. In cases of soft-tissue infiltration, pelvic MRI was additionally obtained. Preoperative imaging assessment always included detailed planning of the surgical approach and the extent of bone and soft-tissue resection. Figure 2 presents pelvic bone tumour radiograms and the most common Enneking resection types with 3D custom-made endoprostheses reconstructions. The types of resection, classified according to Enneking and Dunham, are presented in Table 1.

**Figure 3:**
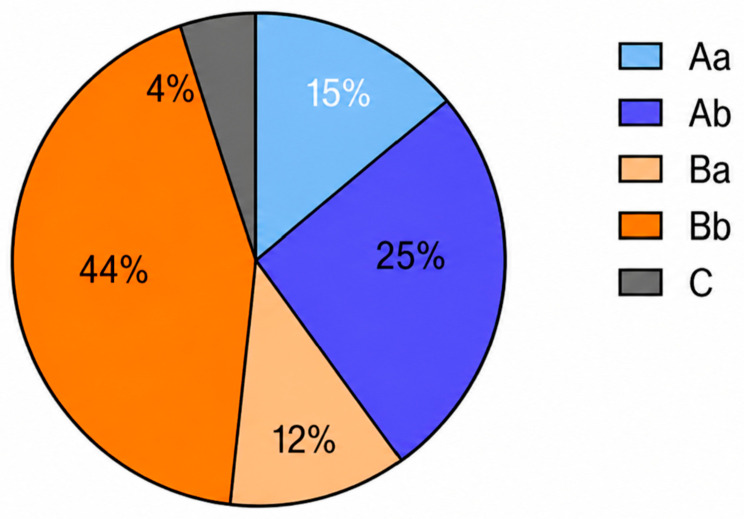
Reconstruction according to the West China Classification.

The reconstruction method was classified according to the West China Classification; see Figure 3.

Postoperatively, pain intensity was assessed using the VAS on postoperative day 7, as well as at 6 weeks, 3 months, and 6 months after surgery. The times to initiation of rehabilitation and ambulation, as well as the types of orthopaedic assistive devices, were evaluated. The range of passive and active joint motion was assessed, with particular attention to the function of individual muscle groups. Patient functional status was evaluated using the HHS and MSTS-93 on day 7, at 6 weeks, and at 3 and 6 months after surgery. The minimal clinically important difference (MCID) was not evaluated. Surgical margins were evaluated based on histopathological findings.

Follow-up examinations were conducted at 6 weeks, 3 months, and 6 months postoperatively, including assessment of radiographic signs of local tumour recurrence. Implant stability was also assessed, including prosthesis osseointegration with bone and signs of implant loosening or damage. Radiological assessment was performed jointly by a senior radiologist and an orthopaedic surgeon. Signs of radiographic osseointegration included absence of radiolucent lines > 2 mm, no implant subsidence, and presence of radial trabeculae of bone extending all the way to the metal component surface. Signs of radiographic implant loosening included implant migration, implant or bone fracture, and presence of radiolucent lines > 2 mm.

The number and type of surgical complications were evaluated, with particular emphasis on endoprosthesis dislocations, postoperative wound-healing problems, infections, bone and implant damage, and injuries to pelvic organs, vessels, and nerves. Wound-healing problems were defined if wound leakage persisted for more than 3 days (≥4). Deep infections were recognized if one of the two big Musculoskeletal Infection Society (MSIS) criteria were satisfied: leakage from the wound with two positive cultures of the same organism or a sinus tract communicating with the prosthesis. Data from all centres were analysed together to reduce centre-specific bias.

Technical abbreviations were defined upon first use. A non-blinded assessment of outcomes was performed. Listwise deletion was applied because missing data were minimal (17 cases). This allowed the analysis to be conducted on a consistent dataset while maintaining clarity and comparability across results. All variables within the groups were assessed for distribution. For normally distributed variables, means and standard deviations are reported; for non-normally distributed variables, medians with upper and lower quartiles are provided. Between-group comparisons were performed using the Kruskal–Wallis test for continuous variables and the chi-square or Fisher’s exact test for categorical variables. Paired *t*-tests were used for repeated measures. A *p*-value of <0.05 was considered statistically significant. All statistical analyses were performed using Statistica version 13 (TIBCO Software Inc., Palo Alto, CA, USA).

## 4. Results

Surgical approaches were selected according to the extent of resection and the reconstruction method. Type I + II internal hemipelvectomies were performed using an iliofemoral approach, whereas Type II + III and complete internal hemipelvectomies were performed using an ilioinguinal approach. In seven cases, an additional incision was made along the lateral border of the rectus femoris muscle. The dressings were inspected on the first postoperative day. For hemipelvectomy procedures, extended antibiotic prophylaxis was administered for 3 days. A second-generation cephalosporin (cefuroxime) was administered 30 min before the skin incision at a dose of 1 g in patients weighing <80 kg and 2 g in those ≥80 kg. The dose was repeated after 3 h if the procedure was prolonged. In the postoperative period, clindamycin was administered at a dose of 600 mg twice a day until wound discharge stopped. In cases where infection was diagnosed, antibiotic therapy was adjusted according to the antibiogram.

The surgical results are included in Table 2. Surgical resection margins were assessed histopathologically and classified as R1 in 12 cases and R0 in 37 cases. All patients were qualified for adjuvant radiotherapy at 6 weeks postoperatively. A single dose of 8 Gy was administered to 34 patients, while the remaining patients received five fractions of 4 Gy. In this study the effect of chemotherapy was not assessed. In 6 cases, postoperative radiographic examinations showed bone defects that were interpreted as local tumour recurrence.

In 16 cases, the operation plan was changed, and not all implant stabilizing elements were used due to poor bone density or sufficient initial stability. In this study, deviations from the surgical plan were not correlated with surgical or functional outcomes.

Intraoperative blood loss ranged from 250 mL to 2300 mL, with a mean of 600 mL. Blood loss was significantly higher in Type II + III and Type I + II + III resections. The mean blood loss was 350 mL for Type I + II resections, 500 mL for Type II + III resections, and 700 mL for Type I + II + III resections.

All patients required a postoperative transfusion of packed red blood cells (PRBCs). The indication for blood transfusion was intraoperative blood loss with clinical symptoms (decreased blood pressure, increased heart rate) or a haemoglobin level < 8 g/dL. Fourteen patients received two units of PRBCs intraoperatively, with transfusion indicated when blood loss exceeded 500 mL. A complete blood count was obtained immediately after surgery in all patients and correlated with intraoperative blood loss.

Postoperatively, the patients received between two and six units of PRBCs, with a mean of three units. When more than four units of PRBCs were transfused, two units of fresh frozen plasma (FFP) were administered.

The duration of surgery ranged from 160 to 260 min, with a mean operative time of 230 min. Procedures involving Type I + II hemipelvectomies were shorter, with a mean duration of 210 min, whereas Type II + III procedures averaged 230 min and Type I + II + III procedures averaged 240 min.

Tranexamic acid (Exacycle) was administered in all cases at a total dose of 2 g, with 1 g given prior to the skin incision and 1 g administered during wound closure.

**Figure 4:**
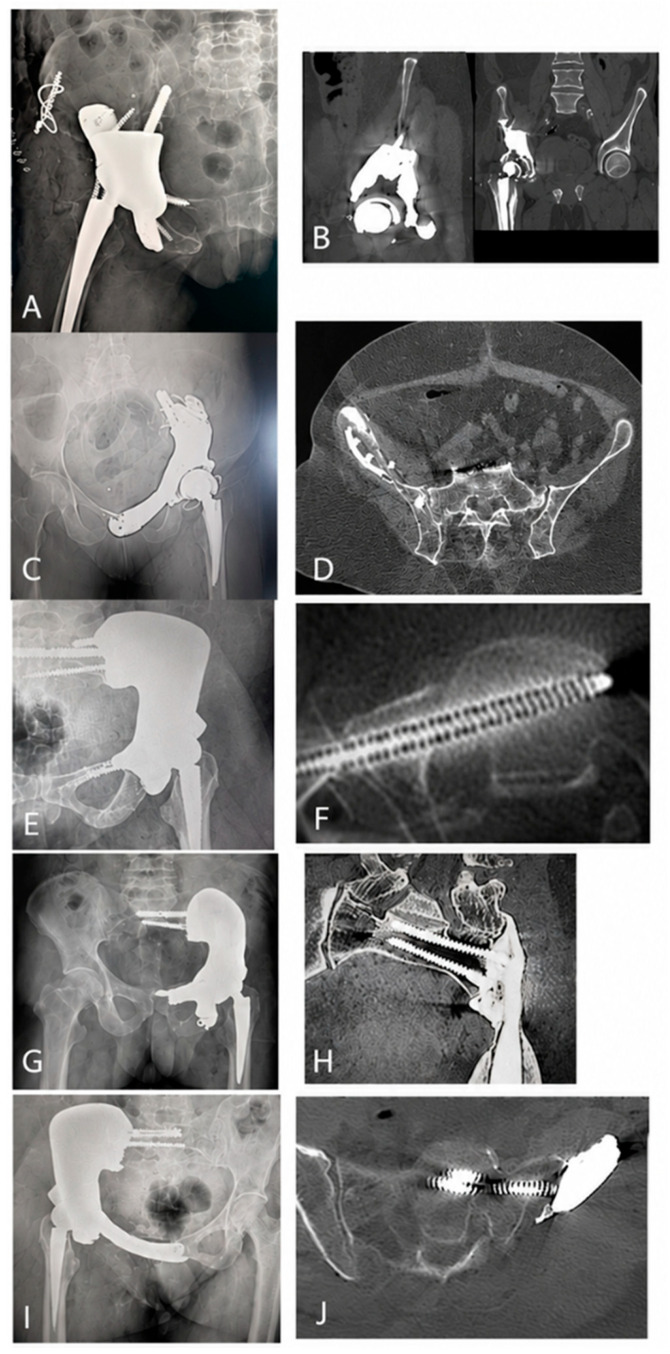
Postoperative radiographs and CT scans obtained at 6 weeks and 6 months, respectively, for each type of pelvic reconstruction according to WChC: WChC-Aa (**A**,**B**), WChC-Ab (**C**,**D**), WChC-Ba (**E**,**F**), WChC-Bb (**G**,**H**), and WChC-C (**I**,**J**).

**Figure 5:**
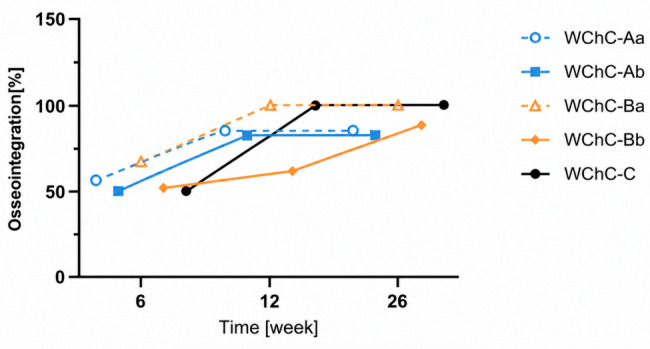
Osseointegration over time after surgery.

The progression of prosthesis osseointegration was assessed on follow-up radiographs during orthopaedic control examinations. Radiographs were acquired in the anteroposterior projection. Pelvic computed tomography was additionally performed at 3 and 6 months postoperatively. The radiographs and CT scans in Figure 4 show details of prosthesis osseointegration. At 6 weeks after surgery, radiological signs of osseointegration were observed in 53% of patients. This increased to 75% and 83% at 3 and 6 months postoperatively, respectively (Figure 5). No implant loosening was observed at 6 weeks. CT scans at 3 and 6 months revealed implant loosening in 7 and 8 cases, respectively.

Surgical complications and radiographic results are presented in Table 3 and Table 4. Prosthesis luxations were observed in 6 patients, all following falls on the floor. Each time, the dislocations were reduced under general anaesthesia without surgery.

In 3 cases of deep infections, we decided to remove the endoprosthesis. These patients were subsequently treated with negative pressure dressing (14–27 days) and received 6 weeks of antibiotic therapy. After wound healing, we did not perform reoperation, and the functional results in these cases were the poorest.

In one case of deep infection, surgical debridement and two weeks of negative pressure dressing with antibiotic therapy resulted in successful wound healing. In all cases, infections were caused by methicillin-resistant *Staphylococcus aureus* (MRSA). In 6 patients with wound-healing problems, wound discharge persisted for 4–9 days (average, 7 days). In all cases, wound cultures were negative, and after negative pressure dressing and antibiotic therapy, the wounds healed.

**Figure 6:**
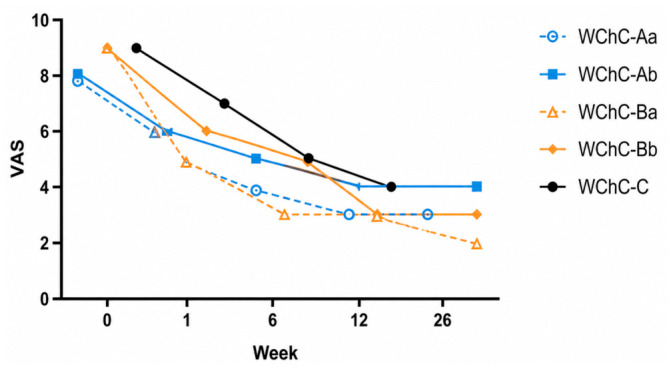
VAS after surgery.

**Figure 7:**
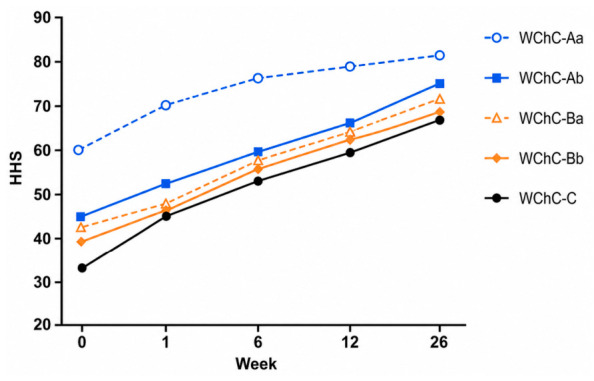
HHS after surgery.

**Figure 8:**
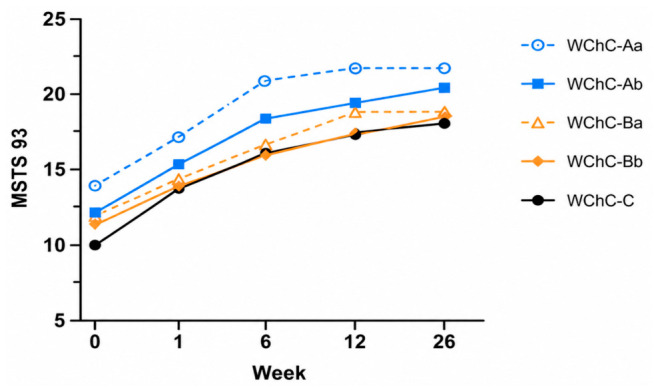
MSTS-93 after surgery.

The functional results are presented in Table 5 and Figure 6, Figure 7 and Figure 8. In all patients, a reduction in pain with a tendency to improve over time was observed. The mean VAS before surgery was 8.5 and decreased to 4.4, 3.4, and 3.2 at 6 weeks, 3 months, and 6 months postoperatively, respectively. Functional assessment revealed significant improvement across all patients. The mean preoperative HHS score was 44; it increased to 64, 70.2, and 76 at 6 weeks, 3 months, and 6 months, respectively. The mean preoperative MSTS-93 score was 12; it increased to 19, 20, and 20.2 at 6 weeks, 3 months, and 6 months postoperatively, respectively.

## 5. Discussion

Only a few studies in the literature have evaluated the use of custom-made 3D-printed pelvic implants in patients following internal hemipelvectomy (IHP) [14]. Individualization of the implant to the patient’s anatomy may enable good surgical and functional outcomes. Precise bone resections and implant positioning increase primary endoprosthesis stability and promote subsequent osseointegration [19,20].

The complex design of modern implants, the use of porous materials mimicking trabecular bone structure, and the incorporation of multiple stabilising elements, such as screws and rods, significantly increase the primary stability of the implant and allow early rehabilitation and weight-bearing ambulation. In addition, patient-specific cutting guides, osteotomy templates, sterile bone models, and intraoperative prosthesis models facilitate and enhance surgical precision. Intraoperative navigation has also been increasingly adopted to further optimise implant positioning [21,22].

Most contemporary 3D-printed implants are manufactured using additive techniques, with porous bone-contact surfaces that resemble the bone microarchitecture, thereby accelerating the osseointegration process [23]. Biomechanical studies have highlighted the importance of reconstructing the pelvic ring and restoring the hip joint centre of rotation, as this ensures balance and reduces the risk of loosening [24,25]. Modern implant designs even allow for stable fixation to the sacrum following complete bone resection.

Early reports on the use of implants in oncologic surgery date back to 1997 and include manually fabricated metal implants fixed to bone with cement, as well as anatomically shaped designs secured using conventional screws. These early solutions were associated with high complication rates, predominantly aseptic loosening (24%), infections (56%), and local tumour recurrence (50%) [26,27]. Currently, many other surgical reconstruction techniques are used, especially impacted bone grafts fixed with metal plates and screws. Protrusio cages and modular prosthesis systems can also be used. The percentage of bone graft healing is in the range of 47–94%, although functional results are unsatisfactory. The use of saddle prostheses has been associated with a high rate of complications (approximately 50%). Next, the use of stemmed cups reduced the total complication rate to 40% and the deep infection rate to 17%, with functional results in MSTS-93 reaching 71%. The inverted ice-cream cone prosthesis further reduced the total complication rate to 37% and the deep infection rate to 11.1%, although the prosthesis luxation rates were high, at 14.8%. The introduction of the LUMIC endoprosthesis reduced the luxation rate to 9%, but the deep infection rate was 28% [1,4,7,8,9,10].

In a systematic review conducted by Brown et al. in 2018, a high overall complication rate across all reconstructive techniques following oncologic hemipelvectomies was confirmed [1]. In patients receiving 3D-printed implants, mechanical complications are most frequently related to improper implant positioning, incorrect placement of stabilizing elements, and dislocations [28].

Infections remain the most common complication following pelvic reconstruction. In the present study, the observed rates of infection, implant loosening, and dislocation should be interpreted with caution due to the relatively short follow-up period and the heterogeneity of the study population. Importantly, complication rates may differ depending on tumour type, extent of resection, patient comorbidities, and perioperative treatment, which were not controlled for in multivariate analysis.

Schenkow et al. identified operative time and the extent of resection as key risk factors for infection [29]. Hilmann et al. reported infection rates of up to 40%, although specific risk factors could not be clearly identified [30].

Angelini et al. demonstrated an increased risk of infection in older patients, those with a high BMI, patients undergoing chemotherapy and radiotherapy, and those with advanced tumours. Additional risk factors include prolonged operative time, blood transfusions, vascular injury, and the extent of resection [9,20]. In the present study, postoperative wound-healing complications occurred in 20% of patients, including deep infections in four patients and delayed wound healing in six cases. The lack of detailed adjustment for these confounding variables represents an important limitation of the current analysis and may partially explain variability in complication rates observed between subgroups.

Apffelstaedt et al. have reported a mean hemipelvectomy time of 7.5 h, with an average blood loss of 3.2 L [31]. In our study, the mean operative time was 230 min, with the longest duration observed in Type I + II + III resections (240 min). Mean blood loss was 600 mL and was likewise highest in Type I + II + III hemipelvectomies. Most researchers have identified internal iliac artery injury as a factor increasing the risk of skin necrosis and infection [28].

Angelini et al. reported favourable radiographic and functional outcomes in patients treated with 3D-printed prostheses, with a mean MSTS score of 64.5%, ranging from 57% to 70% [9,20].

Functional outcomes are strongly influenced by implant design and manufacturing, material properties, and the stability provided by screws and stems that enable biological fixation. Precise implant positioning and primary stability are therefore crucial [10,17,18].

In our study, local tumour recurrence was observed in six (12%) patients. Radiographic evidence of implant osseointegration was present as early as 6 weeks in 53% of patients, increasing to 75% and 83% after 3 and 6 months, respectively. The most common mechanical complications were prosthesis luxations (12%) and implant loosening, observed radiographically at 3 and 6 months postoperatively in 7 (14%) and 8 (16%) patients, respectively. In three cases, implant removal was required due to deep infection. It should be emphasised that these outcomes reflect early postoperative results, and longer-term follow-up is required to assess implant durability, late complications, and oncological outcomes. The discrepancy between the reported overall follow-up duration and the analysed time points should be acknowledged when interpreting these findings. Biomechanical studies by Tile et al. demonstrated that posterior pelvic structures account for 60% of implant stability, while anterior structures account for 40%**.** Accurate implant seating and stable fixation are essential to prevent abnormal force distribution, which may cause loosening or fracture of screws and stems [32]. Park et al. reported very good functional outcomes—namely, an MSTS score of 100%—in patients who received 3D-printed prostheses following Type III hemipelvectomy [33].

Ji et al. reported a mean MSTS score of 57.2% following Type II hemipelvectomy without complete reconstruction of the pelvic ring using first-generation prostheses. Functional outcomes improved to a mean MSTS score of 84% following the introduction of second-generation 3D-printed prostheses with porous titanium interface enhancing bone–implant contact [34].

In our study, postoperative pain decreased substantially. The mean preoperative pain intensity was 8.5 on the VAS, decreasing to 6 at 7 days after surgery, and further to 4.5 and 3.5 at 6 weeks and 3 months, respectively. Functional outcomes were comparable to those reported in the literature. However, the clinical relevance of these improvements should ideally be interpreted in the context of minimal clinically important differences (MCID), which were not assessed in this study. Therefore, the magnitude of functional improvement should be interpreted cautiously from a clinical perspective.

The mean HHS improved from 44 preoperatively to 64 at 6 weeks, 70.2 at 3 months, and 76 at 6 months, with further improvement expected over time. MSTS-93 values increased from a preoperative mean of 12 to 19, 20, and 20.2 at 6 weeks, 3 months, and 6 months, respectively.

Complete reconstruction of the pelvic ring provides good functional outcomes, as demonstrated by Wang et al., who reported a mean MSTS score of 90% with no implant loosening or infections [17].

Application of custom-made prostheses is related to a relatively long design time of about 2 weeks and final implant execution time of about 6–8 weeks. This is especially important in patients with bone sarcomas, as tumour growth may change the extent of required resection, potentially affecting the precision of prosthesis insertion and fixation. The cost of 3D custom-made prosthesis production varies depending on complexity and the manufacturing company. In Poland, the cost ranges from 15 to 70 thousand euros. These practical aspects, including cost, production time, and surgical complexity, should be considered when evaluating the overall clinical utility of custom-made implants, particularly in comparison to alternative reconstruction techniques.

This study is also subject to several sources of bias. As a retrospective analysis, it is prone to selection bias, particularly in patient qualification for custom-made implants. Measurement bias may also be present, especially in the radiographic assessment of osseointegration and implant loosening. Additionally, the multicentre design introduces potential centre-related variability in surgical technique, perioperative management, and outcome assessment. Furthermore, the absence of multivariate analysis and adjustment for confounders limits the ability to draw causal inferences regarding the relationship between surgical technique, implant design, and clinical outcomes. Therefore, the observed associations between the use of 3D-printed implants and favourable functional or radiographic outcomes should be interpreted cautiously and should not be considered as evidence of superiority over other reconstructive methods.

## 6. Conclusions

The use of the commonly accepted Enneking classification to define the resection type, as well as the WChC system to determine the type of pelvis reconstruction, helps to standardize and directly compare study outcomes reported by different centres.

The extent of pelvic tumour resection and the reconstruction method appear to influence surgical parameters, risk of complications, and operative time. The greatest blood loss and longest operative times were observed in patients undergoing total hemipelvectomy (Enneking I + II + III).

Functional outcomes measured using the VAS, HHS, and MSTS-93 scales showed improvement following surgical treatment; however, these findings should be interpreted with caution in the context of the relatively short follow-up period and the lack of assessment of minimal clinically important difference (MCID).

The number of patients demonstrating implant osseointegration increased during the early postoperative period, although longer follow-up is required to assess the durability of these findings.

The use of 3D-printed implants may facilitate precise reconstruction and enable early mobilization and rehabilitation; however, the observed associations should not be interpreted as evidence of superiority over other reconstructive methods.

## 7. Limitations of the Study

The study has several significant limitations. It is a retrospective study and concerns a relatively rarely performed procedure, which resulted in a small sample size. Oncological results, especially overall patient survival, were not analysed. Additionally, there was no control group in the study. A further limitation is that centre-level effects were not assessed. Since participants may have been clustered within centres, unmeasured differences between centres could have influenced the results. Future research should account for this potential clustering using multilevel or mixed-effects modelling.

## Figures and Tables

**Table 1:** Types of resections according to the Enneking classification.

Cancer	Type I + II	Type II + III	Type I + II + III
Others	7	7	1
Renal cancer	3	2	
Breast cancer	9	8	2
Lung cancer	2		
Thyroid cancer	4	1	
Plasmocytoma	2	1	
Total	27	19	3

**Table 2:** Selected parameters assessed during hospitalization of the patients after hemipelvectomy.

	Type I + II	Type II + III	Type I + II + III	*p*-Value
Surgical time [min]	210 (160–230)	230 (180–250)	240 (220–260) *	0.01
Blood loss [mL]	350 (250–700)	500 (250–700)	700 (500–2300) *	0.01
Blood transfusion [blood unit]	2.2 (2–4)	2.6 (2–4)	4 (2–4) *	0.04
Surgical margins R1	5 (19%)	7 (36%) *	0	0.02
Local recurrence	2 (7%)	3 (16%) *	1 (33%)	0.03
Wound healing problems	2 (7%)	3 (16%) *	1 (33%)	0.03
Deep infections	1 (4%)	2 (11%) *	1 (33%)	0.2

Data are presented as mean ± standard deviation, median (lower and upper quartiles), or number (percentage), where appropriate. Asterisks indicate statistically significant differences between groups.

**Table 3:** Surgical complications assessed in the perioperative period.

	WChC–Aa	WChC–Ab	WChC–Ba	WChC–Bb	WChC–C	*p*-Value
Wound-healing complications	0	3 (25%) *	0	2 (10%)	1 (50%)	0.04
Deep infections	0	2 (17%) *	0	1 (5%)	1 (50%)	0.04
Implant loosening:						0.03
6 weeks	0	0	0	0	0	
3 months	1 (14%)	2 (16%)	0	4 (19%)	0	
6 months	1 (14%)	2 (16%)	0	5 (24%) *	0	
Prosthetic dislocations	1 (14%)	2 (16%)	2 (33%) *	1 (5%)	0	0.03
Periprosthetic fractures	0	0	0	0	0	NS
Implant loss	0	2 (16%)	0	1 (5%)	0	NS

Data are presented as numbers (percentages). Asterisks indicate statistically significant differences between groups.

**Table 4:** Radiographic parameters assessed in the perioperative period.

	WChC–Aa	WChC–Ab	WChC–Ba	WChC–Bb	WChC–C	*p*-Value
Osseointegration:						NS
6 weeks	4 (57%)	6 (50%)	4 (66%)	11 (50%)	1 (50%)	
3 months	6 (86%)	10 (83%)	6 (100%)	13 (59%)	2 (100%)	
6 months	6 (86%)	10 (83%)	6 (100%)	17 (77%)	2 (100%)	

Data are presented as numbers (percentages).

**Table 5:** Functional outcomes after custom-made prosthetic reconstruction.

	WChC-Aa	WChC-Ab	WChC-Ba	WChC-Bb	WChC-C	*p*-Value
VAS—before (CI 95%)	8 (7.6–8.4)	8 (7.6–8.5)	9 (8.6–9.4)	9 (7.5–9.4)	9 (8.5–9.8)	
VAS—6 months	3 * (2.7–3.2)	4 * (3.6–4.3)	2 * (1.8–2.3)	3 * (2.8–3.2)	4 * (3.8–4.2)	0.01
HHS—before	60 (55–64)	45 (42–48)	42 (39–46)	40 (38–44)	36 (33–41)	
HHS—6 months	84 * (80–88)	76 * (73–77)	76 * (74–78)	72 * (68–78)	72 * (67–79)	0.01
MSTS-93—before	14 (12–14)	12 (12–16)	12 (11–16)	12 (11–14)	10 (7–12)	
MSTS-93—6 months	22 * (19–25)	21 * (19–23)	20 * (18–22)	19 * (17–21)	19 * (17–22)	0.01

Data are presented as numbers (percentages). Asterisks indicate statistically significant differences between groups.

## Data Availability

All data generated or analysed during this study are included in this manuscript.

## Abbreviations

VAS	Visual Analogue Scale
HHS	Harris Hip Score
MSTS-93	Musculoskeletal Tumor Society
WChC	West China Classification
PRBCs	Packed Red Blood Cells
MSIS	Musculoskeletal Infection Society
FFP	Fresh Frozen Plasma
FPI	Focus Primarius Ignotus
STROBE	Strengthening The Reporting Of Observational Studies in Epidemiology
MCID	Minimal Clinically Important Difference
